# The impact of migrant work experience on rural households’ participation in digital finance: Evidence from China

**DOI:** 10.1371/journal.pone.0337525

**Published:** 2025-11-21

**Authors:** Fan Xu, Xiaolei Zhang, Lezhu Zhang

**Affiliations:** School of Economics and Management, South China Agricultural University, Guangzhou, China; University of Florida, UNITED STATES OF AMERICA

## Abstract

Currently, the development of digital finance in China’s rural areas remains in its early stages. The proportion of rural households participating in digital finance and the types of participation are relatively low. This hinders the realization of digital finance’s inclusive effects. This paper examines the influence of migrant work experience on participation in digital financial of rural households using data from the China Household Finance Survey (CHFS). The findings suggest that migrant work experience significantly increases the probability of rural households’ digital financial participation and increases the breadth of participation. Heterogeneity analysis finds that migrant work experience promotes digital financial participation more for rural households with migrant formal work experience, migrant entrepreneurial experience, completion of compulsory education, and long-tail groups. Mechanism analysis finds that migrant work experience can influence rural households’ digital financial participation by improving their financial literacy and broadening their social networks.

## 1. Introduction

Along with the rapid development of information and communication technologies such as the Internet and big data, finance has combined with them to form a new financial business model. Compared to traditional finance, digital finance significantly lowers the barriers to financial services and transaction costs while expanding the reach of financial products [1]. Digital finance provides financial services to underserved populations globally to promote their consumption [2], entrepreneurship [3,4], household welfare [5], and regional economic development [6]. Especially for rural areas beyond the reach of traditional finance, digital finance breaks the geographical limitation of financial services, alleviates financial exclusion and poverty in remote rural areas, and becomes an important way to promote the development of inclusive finance [7,8].

Family digital finance participation refers to the use of digital financial tools such as digital payments, digital wealth management, and digital lending by households. Despite the many benefits of digital finance, the participation rate of Chinese rural households in digital finance is generally low [9]. 2017 China Household Finance Survey (CHFS) data show that the proportion of rural households participating in digital finance was only 20.96% [10]. 2019 Rural survey data from four Chinese provinces show that less than 20% of rural households use more than two digital financial products [11]. 2021 Data from the China Rural Economy and Rural Finance Survey (CRERFS) similarly show that the average number of rural households using three types of digital finance is only 1.025 [12]. In fact, the use of digital finance puts forward higher requirements for farmers’ digital skills and financial knowledge, which makes rural residents with backward economy and low average education level more likely to be excluded [13]. The ability of rural households to participate in digital finance is key to enabling rural households to enjoy the dividends of digital experience development, and improving the well-being of rural residents. Therefore, research on the factors influencing rural households’ digital financial participation has recently attracted increasing attention from scholars and policymakers in developing countries.

Existing literature has primarily examined rural households’ participation in digital finance from perspectives including personal financial literacy [14], ICT usage [9], e-commerce adoption [15], regional digital financial development [16], regional network infrastructure [17], and regional pandemic situations [18]. Economists have accumulated a large body of evidence that an individual’s early experiences can have a significant impact on later behavioral decisions. Studies by sociologists and psychologists have found that an individual’s early experiences can have an impact on later decision-making and behavioral competencies [19,20]. First, early experiences have a profound effect on an individual’s later decisions. For example, a manager’s experience can have an impact on his or her later managerial decisions in the firm. CEOs’ international work experience leads to higher compensation [21]. Firms whose CEOs experienced the Chinese famine early in their careers experienced lower stock price declines than firms that did not [22]. Second, early experiences can influence an individual’s ability to behave later in life. For example, work experience can promote pro-social behavior in adolescents [23]. Early grassroots work experience for tax officials enhances their ability to serve as Chinese officials later in life [24]. As one of the significant life experiences for farmers, migration work experience inevitably exerts a considerable influence on their decision-making. However, no literature has yet explored this phenomenon.

This paper makes the following key contributions. First, it explores the impact of migrant work experience on rural households’ participation in digital finance, thereby enriching the research perspective. Second, it reveals the underlying mechanisms through which migrant work experience influences rural households’ digital finance participation, deepening our understanding of the intrinsic link between migrant work experience and digital finance engagement. Finally, it examines the heterogeneous effects of migrant workers’ work experience on rural households’ digital finance behaviors, providing insights for developing differentiated policies to enhance rural digital finance participation.

The rest of the paper is organized as follows: section 2 provides a theoretical analysis of the impact of migrants’ work experience on the financial vulnerability of rural households and formulates the research hypotheses. Section 3 presents the data, models, and variables used in the empirical study. Section 4 reports the baseline results, robustness tests, and heterogeneity analysis results of the empirical study. Section 5 presents the results of mechanism analysis. Section 6 presents the conclusions and corresponding policy recommendations.

## 2. Theoretical analysis and research hypotheses

Under the influence of urban human capital externalities, migrant workers subconsciously mimic and learn from high-skilled individuals, achieving complementary advantages in skills and resources. This enhances their income, cognitive and non-cognitive abilities, and accumulates human capital, ultimately making their labor experience a key event in their lives. This migrant work experience forms a special kind of memory imprint that retains previous psycho-cognitive patterns and behavioral habits even after the labor force returns home, and has a profound impact on the family’s economic behavioral decisions. It has been shown that migrant work experience positively contributes to farmers’ economic capital, human capital, social capital, and visionary attitudes [25,26], thereby contributing to rural households’ participation in digital finance. Therefore, this article proposes the following hypothesis:

**Hypothesis 1:** Migrant work experience can facilitate rural households’ participation in digital finance.

Migrant work experience can facilitate rural households’ participation in digital finance by increasing their level of financial literacy. Financial literacy, as a vital form of human capital, effectively promotes household participation in digital finance by enhancing families’ understanding of financial products and decision-making capabilities [27,28]. After migrating to economically developed cities for work, farmers gain significantly increased access to financial institutions and products such as banks, securities, credit cards, and funds. This exposure continuously broadens their financial horizons and market awareness, ultimately fostering the accumulation of financial knowledge [29]. Moreover, migrant work contributes to increased household income. As earnings rise, farmers develop greater needs for wealth management and asset appreciation, thereby motivating them to proactively acquire financial knowledge [30]. This, in turn, enhances their ability to manage household finances and preserve or grow their assets.

The experience of migrant work can facilitate rural households’ participation in digital finance by broadening their social networks. Social networks, as an important informal system, can establish a demonstration effect of rural households’ participation in digital finance [31]. Within social networks, when acquaintances utilize digital financial products and derive benefits from them, they share various information during daily interactions. This not only helps other households broaden their access to information, enabling them to better understand digital finance, but also builds trust in digital financial platforms by disseminating experiences. This reduces feelings of unfamiliarity and apprehension, thereby stimulating willingness to participate in digital finance. Furthermore, social networks foster mutual assistance among members. When encountering difficulties or risks during digital financial activities, individuals can seek advice and support from friends, relatives, and others within their relational networks [32]. This reduces their perceived risks associated with digital finance participation and strengthens their confidence to engage. Migrant work not only allows rural families to maintain their original kinship and geographic networks, but also expands new social networks such as karma and friendships in the new work [33]. Therefore, this article proposes the following hypothesis:

**Hypothesis 2**: Migrant work experience can facilitate rural households’ participation in the digital finance by increasing their financial literacy and expanding their social networks.

## 3. Materials and methods

### 3.1. Data

The data used in this study come from the China Household Finance Survey (CHFS), which is conducted by the China Household Finance Survey and Research Center of Southwestern University of Finance and Economics on a sample basis every two years, and comprehensively tracks households’ dynamic financial behaviors, providing the researcher with detailed information on household credit, financial assets, and insurance. The data used in this study are from the 2019 China Household Finance Survey (CHFS). The CHFS data sample covers 29 provinces (autonomous regions and municipalities), 345 counties, and 1,359 communities (villages) nationwide, with a stratified sample of 34,643 households, ensuring strong representativeness. In addition to the demographic characteristics, economic features, and financial literacy information, the 2019 CHFS data also includes household migration and migrant work experiences, as well as participation in digital finance. According to the purpose of the study, the sample of heads of households under 18 years old and those who go out to school are excluded, and the sample data of 9096 rural households are retained after removing the missing values. The per capita GDP data at the regional level comes from the China Statistical Yearbook.

### 3.2. Model

This article examines the impact of migrant work experience on both the probability and breadth of digital financial participation among rural households. Since the explanatory variable whether rural households participate in digital finance is a binary discrete variable, a probit model is constructed. The model is set up as follows:


$$P(Y=1|x)=\Phi (\alpha_{0}+\alpha_{1}Experience_{i}+\lambda X_{i}+\varepsilon_{i})$$ (1)

Where P(Y=1|x) is the probability of rural households’ digital financial participation, $Experience_{i}$ denotes whether householder has migrant work experience. $X_{i}$ is a vector of control variables, and $\varepsilon_{i}$ is the error term.

Since the explanatory variable breadth of household digital finance is a discrete variable with counting characteristics, this paper constructs a Poisson model to analyze the impact of migrant workers’ work experience on the breadth of farmers’ participation in digital finance. The hypothesis probability is determined by the Poisson distribution of parameters, and the model is set up as follows:


$$P(Y_{i}=y_{i}|x_{i})=\frac{e^{-\lambda_{i}}\lambda_{i}^{y_{i}}}{y_{i}!}$$ (2)

Where $P(Y_{i}=y_{i}|x_{i})$ is the number of households using digital financial instruments, $\lambda_{i}$ is Poisson’s arrival rate, and represents the breadth of farmers’ participation in digital finance.

Furthermore, based on the preceding analysis, farmers’ financial literacy and social networks mediate the impact of migrant work experience on their participation in digital finance. Therefore, this article constructs a mediation model to test this. The traditional mediation model does not consider the endogeneity of the mediating variables, and when the mediating variables have endogeneity problems, resulting in the estimation results may be biased. The mediating variables in this article are financial literacy and social networks of farm households, both of which have endogeneity problems such as omitted variables or reverse causality that may exist between them and digital financial participation of rural households. When the intervening variable and the mediating variable are endogenous at the same time, the instrumental variables of the intervening variable and the instrumental variables of the mediating variable need to be utilized to identify the mediating effects. This article builds on the following model [34] by adding instrumental variables for the mediating variables to complete the causal identification of mediating effects:


$$M_{i}=\beta_{0}+\beta_{1}Experience_{i}+\beta_{2}X_{i}+\omega_{i}$$ (3)

$$Y_{i}=\delta_{0}+\delta_{1}Experience_{i}+\delta_{2}M+\delta_{3}X_{i}+\nu_{i}$$ (4)

In model (3) $M_{i}$ are channel variables, including financial literacy and social network. $\beta_{1}$ denotes the effect of migrant working experience on each channel variable, $\omega_{i}$ is the error term; $Experience_{i}$ is the only endogenous variable in the model. In model (4), $\delta_{1}$ denotes the direct effect of migrant working experience on rural households’ digital financial participation; $\delta_{2}$ denotes the effect of channel variables on rural households’ digital financial participation after controlling for the migrant work variable, $\nu_{i}$ is the error term; The model includes two endogenous variables, $Experience_{i}$ and channel variables.

### 3.3. Description of focus variables

#### 3.3.1. Explained variable.

This paper has two explanatory variables: probability of participation in digital finance (Probability of participation in digital finance) and breadth of participation in digital finance (Breadth of Participation in Digital Finance). Based on the definition of digital finance by existing scholars, it mainly refers to traditional financial institutions providing payment, financing, investment and other new types of financial services with the help of digital technology [1]. Therefore, this paper focuses on measuring rural households’ digital finance participation by examining the use of the most frequently used financial instruments such as digital payment, digital lending, and digital finance. The questions on digital payment, digital lending, and digital finance management in the questionnaire used are (1) Digital payment: have households used payment methods such as online banking, mobile banking, Alipay, and WeChat? (2) Digital lending: have households used internet borrowing, credit loans or crowdfunding? (3) Digital finance: Have they purchased financial products? The variable of whether to participate in digital finance and the variable of the breadth of participation in digital finance are measured as follows: if a rural household has used any one of the financial instruments such as digital payment, digital lending, digital finance, etc., it is considered to have participated in digital finance, which takes the value of 1, and vice versa 0. The breadth of participation in digital finance is measured by the number of types of digital financial instruments mentioned above that have been used by the rural household.

#### 3.3.2. Core explanatory variables.

The core explanatory variable in this article is the migratory work experience of the farm household, which takes the value of 1 if the head of the household has lived or worked for more than half a year outside of the province/city where he/she resides, and 0 otherwise. In addition, since this article focuses on work experience, the sample of those who have migrated due to their schooling has been excluded.

#### 3.3.3. Intermediary variable.

The mediating variables for this article are financial literacy and social networks. In the measure of financial literacy, considering the differences in the level of financial literacy represented by farmers answering “I don’t know” and answering incorrectly when confronted with a financial literacy question [35], the number of direct answers to financial questions and the number of correct answers to financial questions were used respectively to measure farmers’ financial literacy level. The three main financial literacy questions in the questionnaire include interest question, inflation question, and compound interest question. The questionnaire used contained questions related to financial literacy in three areas: interest, inflation, and compound interest. Interest problem: Assuming that the bank’s interest rate is 4% per year, if you deposit 100 yuan for 1 year, the principal and interest you will receive after 1 year will be?; Inflation problem: Assuming that the bank’s interest rate is 5% per year and the inflation rate is 8% per year, what will you be able to buy after one year of saving 100 yuan in the bank?; Compound Interest Problem: If you take out a loan or deposit 10,000 yuan and compound interest at 10% per year, what is the total amount of principal and interest you will need to repay after two years?. For each of the above questions, two dummy variables can be generated for whether or not to answer “don’t know” and whether or not to answer incorrectly, and a total of six dummy variables can be obtained for the three questions, and the factor analysis method is used to get the score of financial literacy of the farmers.

In the measure of social network, Rural China is a highly humanized and relational society, and social relationships among people often follow the agreed-upon principle of “exchange of gifts”. Family gift income and expenditure is an important means of maintaining family social network relationships, and can reflect the strength of family social network relationships. Therefore, this article uses the logarithm of the total amount of household gift income and expenditure to measure the level of social networks.

#### 3.3.4. Instrumental variable.

In this study, three variables may exhibit endogeneity: migrant work experience, financial literacy, and social networks. This stems from potential omitted variables related to migrant work experience. Simultaneously, household digital financial participation may also exert a reverse effect on financial literacy and social networks. Households with broader participation are more likely to access financial information online and form new social connections, thereby achieving higher levels of financial literacy and social networks. Therefore, to effectively mitigate estimation biases arising from reverse causality and omitted variables, this study requires establishing corresponding instrumental variables for each endogenous variable, while simultaneously satisfying the requirements of instrument relevance and exclusivity.

First, drawing upon existing literature [36,37], this study selects the migration work atmosphere within the village (community) (IV_mw) as an instrumental variable for rural households’ migration work experience. This is measured by the proportion of households within the village (community) that have migration work experience, excluding the interviewed household. In terms of relevance, given the informational network characteristics of rural household migration work [38], households within the same village (community) share work information and experiences, assisting others in migration efforts. When a village has more migrants, the likelihood of the surveyed household engaging in migration work increases. Regarding exclusivity, the migration work environment within villages does not directly influence household participation in digital finance. This is because the migration work environment itself can only provide information or social pressure. Only when households actually engage in migration work will key factors such as income growth, improved financial literacy, and changes in financial needs be directly triggered, thereby driving the use of digital finance. Without actual migration experience, other channels like idea dissemination or peer modeling often yield limited results due to the absence of economic incentives and foundational capabilities.

Second, this study selects the interaction term between regional traditional financial development levels and household attention to financial information (IV_fl) as an instrumental variable for financial literacy. It measures traditional financial development levels using the logarithm of regional financial institutions’ loan balances in 2014. The choice of 2014 stems from the fact that China’s digital inclusive finance began rapid development only after the State Council issued the “Guiding Opinions on Promoting the Healthy Development of Internet Finance” in 2015. In terms of correlation, regions with more developed traditional financial sectors provide residents with greater opportunities for both passive exposure and active learning of financial knowledge. Rural households with high attention to financial information are more likely to proactively seek out financial news, market trends, wealth management knowledge, and information from their surroundings, thereby achieving higher levels of financial literacy. Regarding exclusivity, prior to the rise of digital inclusive finance in China, the level of traditional financial development could only influence households’ digital financial participation decisions by affecting their financial literacy.

Finally, this study selected the interaction term between the proportion of the region’s most common surname and household financial support provided to non-household members (IV_sn) as an instrumental variable for social networks. In terms of relevance, surnames serve as a key indicator for distinguishing whether rural households belong to the same clan [39]. A higher number of prevalent surnames in a region indicates broader social networks among rural households. Simultaneously, household financial support to non-household members reflects rural households’ capacity for external social interaction, closely linked to expenditures on household social networks. Regarding exclusivity, the proportion of surnames in a village carries distinct clan and cultural characteristics, unaffected by individual household traits. It is unlikely to be influenced by variables other than household social networks, thereby satisfying the exogeneity requirement for the instrumental variable.

#### 3.3.5. Control variable.

This paper selects a series of control variables at the level of personal characteristics of farm households, household characteristics and regional characteristics, respectively. Individual characteristics include sex (Gender), age (Age), years of education (Education), marital status (Marriage), and health (Health) of the household head. Household characteristics variables include owner-occupied housing (House), household size (Size), household Elderly dependency ratio (Elder), household Child dependency ratio (Child), total household income (Income), total household assets (Assets), and business engaged in by the household (Commerce). Regional characteristics variables include the logarithm of GDP per capita (Pergdp) and regional dummy variables. Table 1 shows the definitions of the variables.

**Table 1:** Variables definitions.

Variable	Definition
Probability of participation in digital finance	Whether the family is involved in digital finance: yes = 1; no = 0
Breadth of participation in digital finance	The number of types of financial instruments used by households
migrant working experience	Whether the householder used to be a migrant worker: yes = 1; no = 0
Financial literacy	Calculated using factor scoring method
Social network	The logarithm of the total amount of gifts received and paid by the family during holidays and red and white celebrations
IV_mw	The proportion of rural families in the same village (community) who have experience of working outside the home, except for their own family
IV_fl	The interaction term between the level of traditional financial development in a region and the level of household attention to financial information
IV_sn	The interaction between the proportion of the largest surname in the region and the financial support provided by non family members in the family
Gender	Male = 1, otherwise = 0
Age	The age of the householder in 2019
Education	The year of education of the householder in 2019: no education: 0; primary school: 6; junior high school: 9; senior high school/professional high school: 12; junior college/higher vocational school: 15; undergraduate: 16; master’s degree: 18; doctorate: 22
Marriage	Whether the householder is married: yes = 1; no = 0
Health	Compared with peers, the household head’s physical condition is good or average = 1; not good = 0
House	Whether the family have their own house: yes = 1; no = 0
Size	The number of family members.
Older	The ratio of the number of old people (Over 60 years old) to the total number of people in a family.
Child	The ratio of the number of children (under 14 years old) to the total number of people in a family.
Income	The total annual income (yuan) of the household last year.
Assets	The total asset (yuan) of the household last year.
Commerce	Whether the household engage in industry and commerce: yes = 1; no = 0
Pergdp	The logarithm of per capita GDP in the province in 2019.
East	East = 1, others = 0
West	West = 1, others = 0
Digital financial participation in villages	Average digital financial participation in the village excluding one’s own household.
Rural financial institutions	Proportion of rural financial outlets in the region.
Rural digital infrastructure	Number of rural broadband access households in the region (10,000 households).

## 4. Empirical results

### 4.1. Descriptive statistical results

Table 2 shows the descriptive statistics and mean differences of the main variables. The result of the mean value of rural households’ digital finance participation shows that the proportion of rural households participating in digital finance is 32% and the average breadth of participation in digital finance is 0.368. This indicates that the probability and breadth of digital financial participation are not high. The mean test also revealed that households with migrant work experience exhibit a higher probability and greater breadth of participation in digital finance.

**Table 2:** Descriptive statistics.

Variable	Full Sample			No migrant work experience			Migrant work experience			Mean Difference
	Obs	Mean	Std.	Obs	Mean	Std.	Obs	Mean	Std.	
Probability of participation in digital finance	9096	0.322	0.467	7615	0.291	0.454	481	0.481	0.500	−0.187***
Breadth of participation in digital finance	9096	0.368	0.569	7615	0.332	0.550	481	0.553	0.627	−0.218***
Migrant work experience	9096	0.163	0.369	7615	0	0	1481	1	0	−1.00
Financial literacy	9096	−0.017	0.984	7615	−0.047	0.958	1481	0.139	1.091	−0.175***
Social network	8760	0.205	0.317	7323	0.191	0.305	1437	0.277	0.364	−0.085***
IV_mw	9096	0.006	0.013	7615	0.002	0.006	1481	0.029	0.016	−0.027***
IV_fl	9049	43.046	10.755	7571	43.140	10.779	1478	42.564	10.622	0.576*
IV_sn	9083	0.178	0.117	7603	0.183	0.122	1480	0.149	0.077	0.035***
Gender	9096	0.877	0.329	7615	0.865	0.342	1481	0.938	0.241	−0.073***
Age	9096	54.479	10.951	7615	55.249	10.713	1481	50.521	11.305	4.728***
Education	9096	7.398	3.301	7615	7.242	3.365	1481	8.203	2.814	−0.961***
Marriage	9096	0.894	0.308	7615	0.891	0.312	1481	0.912	0.284	−0.021**
Health	9096	0.763	0.425	7615	0.752	0.432	1481	0.821	0.383	−0.069***
House	9096	0.966	0.182	7615	0.965	0.185	1481	0.971	0.168	−0.006
Size	9096	3.389	1.652	7615	3.349	1.649	1481	3.594	1.650	−0.245***
Child	9096	0.108	0.164	7615	0.102	0.161	1481	0.138	0.177	−0.037***
Older	9096	0.169	0.306	7615	0.175	0.313	1481	0.137	0.267	0.038***
Income	9096	5.273	13.368	7615	5.012	13.919	1481	6.617	9.964	−1.605***
Assets	9096	42.587	92.169	7615	41.396	94.170	1481	48.712	80.860	−7.316***
Commerce	9096	0.088	0.284	7615	0.083	0.276	1481	0.116	0.320	−0.033***
Pergdp	9096	11.004	0.327	7615	11.001	0.331	1481	11.018	0.305	−0.017*
East	9096	0.316	0.465	7615	0.325	0.469	1481	0.268	0.443	0.057***
West	9096	0.348	0.476	7615	0.344	0.475	1481	0.365	0.482	−0.021
Digital financial participation in villages	9095	0.297	0.157	7614	0.295	0.157	1481	0.306	0.159	−0.011**
Rural financial institutions	9098	0.295	0.051	7615	0.295	0.052	1481	0.298	0.048	−0.004**
Rural digital infrastructure	8712	550.719	346.755	7248	544.134	351.071	1463	583.538	322.688	−39.405***

Note:***,** and * indicate significance at the 1%,5% and 10% levels, respectively.

### 4.2. Benchmark regression

Table 3 reports the results of the baseline regressions on the impact of migrant work experience on rural households’ digital financial participation. The results in columns (1) and (3) find that the coefficients on the impact of migrant work experience on the probability and breadth of rural households’ digital financial participation are positively significant at the 1% statistical level without the inclusion of control variables. The results in columns (2) and (4) find that the effect of migrant work experience on both the probability of participation and breadth of participation in digital finance for rural households is also positively significant when all control variables are added. The above results indicate that migrant work experience contributes significantly to both the probability and breadth of digital financial participation of rural households.

**Table 3:** The Impact of Migrant Work Experience on Rural Households’ Digital Financial Participation.

Variable	Participate in digital finance		Breadth of participation	
Probit		Poisson	
(1)	(2)	(3)	(4)
Migrant work experience	0.175^***^ (0.012)	0.085^***^ (0.011)	0.188^***^ (0.013)	0.084^***^ (0.012)
Gender		−0.016 (0.014)		−0.010 (0.019)
Age		−0.011^***^ (0.000)		−0.013^***^ (0.001)
Education		0.017^***^ (0.001)		0.021^***^ (0.002)
marriage		−0.026 (0.016)		−0.024 (0.024)
Health		0.068^***^ (0.011)		0.123^***^ (0.018)
House		0.032 (0.027)		0.064^*^ (0.036)
Commerce		0.214^***^ (0.015)		0.188^***^ (0.013)
Size		0.045^***^ (0.003)		0.062^***^ (0.004)
Child		−0.164^***^ (0.032)		−0.262^***^ (0.038)
Older		−0.146^***^ (0.021)		−0.400^***^ (0.035)
Income		0.001 (0.001)		0.001^*^ (0.000)
Assets		0.000^***^ (0.000)		0.000^***^ (0.000)
Pergdp		0.046^***^ (0.016)		0.083^***^ (0.020)
East		0.016 (0.012)		0.012 (0.015)
West		−0.007 (0.010)		−0.016 (0.014)
N	9096	9096	9096	9096
Pseudo R^2^	0.017	0.238	0.011	0.141

Note: The coefficient is the marginal effect. ***,** and * indicate significance at the 1%,5% and 10% levels, respectively. The standard errors are clustered at the household level and are reported in parentheses.

Among the control variables, the age, education level, and health status of the household head significantly influence rural households’ participation in digital finance. At the household level, household size, business investment, and total assets positively affect households’ digital finance behavior. At the regional level, per capita GDP positively influences households’ digital finance behavior. The household head’s gender, marital status, owner-occupied housing, and total income do not significantly affect households’ digital finance behavior.

### 4.3. Endogeneity checks

Although there is no reverse causality between past migrant labor experiences of rural households and current digital financial participation of rural households, the results may still be biased by omitted variables. Table 4 reports the results of the estimation using instrumental variables. Column (1) presents the estimation results using the IV-Probit model. The results show that the coefficient of the effect of out-of-home labor experience on the probability of digital financial participation of rural households is positively significant at the 1% statistical level. Column (2) presents the estimation results using IV-Poisson model. The results show that the coefficient of the effect of migrant labor experience on the breadth of digital financial participation of rural households is positively significant at the 1% statistical level. The F-value of 325.37 in the first stage indicates that the selected instrumental variable is not a weak instrumental variable. The results of the endogeneity test show that after considering endogeneity, the migrant work experience still has a positive impact on the probability of rural households’ digital financial participation and the breadth of participation.

**Table 4:** Endogeneity checks results.

Variables	Participate in digital finance	Breadth of participation
IV-Probit	IV-Poisson
(1)	(2)
Migrant work experience	0.432*** (0.052)	0.358*** (0.044)
Control variables	YES	YES
Constant	−1.411** (0.649)	−3.042*** (0.608)
First stage F value	325.37	
N	9096	9096

Note: ***,** and * indicate significance at the 1%,5% and 10% levels, respectively. The standard errors are clustered at the household level and are reported in parentheses.

### 4.4. Robustness checks

#### 4.4.1. Propensity score matched.

Since the migrant work decision-making is not random, but affected by many factors such as individual farmers and households, it may lead to endogenous problems caused by sample self-selection, resulting in biased results. To solve this problem, this paper use the propensity score matching method (PSM) to mitigate this bias. The propensity score method (PSM) could create a near-randomized experiment by identifying the sample that is closest to the treatment and control groups in terms of covariates, in order to reduce estimation bias. In this study, according to whether the farmers have migrant work experience, the sample was divided into families with migrant work experience (treatment group) and families with no migrant work experience (control group).By using the logit model to calculate the propensity score with Participate in digital finance and Breadth of participation as the dependent variables, four common matching methods, namely nearest neighbor matching (K = 3), radius matching, kernel matching and local linear regression matching, were selected to re-estimate the impact of migrant work experience on rural households’ digital financial participation. The results of the balance test (S1 Table) show that none of the P-values were significant after matching, indicating that there was no significant difference between the treatment and control groups.

Table 5 reports the results of the average treatment effect (ATT) of migrant work experience on the probability of digital financial participation and the breadth of participation of rural households estimated using the PSM. The estimation results of the four matching methods are less different, but all are positively significant at the 1% level. The average treatment effect of outwork experience on the probability of rural households’ digital financial participation ranges between 0.085 and 0.102, and the average treatment effect of outwork experience on the breadth of rural households’ digital financial participation ranges between 0.101 and 0.122. The PSM estimation results are relatively close to the baseline regression results, which further validates the robustness of the main findings.

**Table 5:** Average Treatment Effect (ATT) results.

	Probability of participation in digital finance				Breadth of participation in digital finance			
Matching methods	Treatment group mean	Control group mean	ATT	T value	Treatment group mean	Control group mean	ATT	T value
Nearest-Neighbor Matching	0.481	0.396	0.085^***^	5.17	0.552	0.451	0.101^***^	4.91
Radius Matching	0.481	0.388	0.093^***^	6.41	0.552	0.439	0.112^***^	6.24
Nuclear matching	0.481	0.379	0.102^***^	7.11	0.553	0.431	0.122^***^	6.82
Local linear matching	0.481	0.388	0.093^***^	4.70	0.552	0.441	0.111^***^	4.56

Note: ***,** and * indicate significance at the 1%,5% and 10% levels, respectively.

#### 4.4.2. Alternative data.

While the instrumental variable method was used in the previous section to mitigate potential omitted variable problems, it was considered that the use of cross-sectional data also introduces omitted variable bias that may lead to endogeneity problems.

To further overcome potential endogeneity problems, this paper will construct balanced panel data for estimation using Chinese household finance data (CHFS) for 2017 and 2019, and the descriptive statistics of the data are shown in S2 Table, and the regression results are shown in Table 6. This paper controls for the same control variables as the baseline regression, except for fixing time and region. The results report that the effect of migrant work experience on both the probability and breadth of digital financial participation of rural households is positive and significant, indicating that migrant work experience has a significant contribution to digital financial participation of rural households.

**Table 6:** Using panel data results.

Variable	Probability of participation in digital finance	Breadth of participation in digital finance
IV-Probit	IV-Poisson
(1)	(2)
Migrant work experience	0.517*** (0.076)	0.499*** (0.078)
Control variables	YES	YES
Time fixed effects	YES	YES
Province fixed effects	YES	YES
Constant	−3.200 (2.678)	−5.332 (3.584)
First stage F value	19.19	
N	9796	9796

Note: ***,** and * indicate significance at the 1%,5% and 10% levels, respectively. The standard errors are clustered at the household level and are reported in parentheses.

#### 4.4.3. Instrument Variable Exclusivity.

The exclusivity condition for the instrumental variable of village migration work atmosphere may not be satisfied. While migration work atmosphere can influence digital financial participation through the migration decisions of surveyed households, this may not be the sole pathway. For instance, under peer group effects, the migration work atmosphere within the village exerts the same influence on migration decisions of households other than the surveyed one, with migration experience directly impacting their digital financial participation. In other words, the instrumental variable may indirectly influence the respondent household’s digital financial participation by affecting other households’ engagement. Concurrently, an infrastructure effect may exist: villages with stronger migration work atmospheres may attract more financial institutions and digital infrastructure, thereby enhancing household digital financial participation.

Given these considerations, this study incorporates village digital financial participation, rural financial institutions, and rural digital infrastructure as control variables in the model to block these pathways and enhance the exclusivity of the village migration work atmosphere as an instrumental variable. Table 7 shows that after adding these controls, the impact of migration work experience on rural households’ digital financial participation remains robust.

**Table 7:** Instrument variable exclusivity results.

Variable	Probability of participation in digital finance	Breadth of participation in digital finance
IV-Probit	IV-Poisson
(1)	(2)
Migrant work experience	0.420*** (0.054)	0.341*** (0.045)
Digital financial participation in villages	0.748*** (0.107)	0.620*** (0.095)
Rural financial institutions	0.347 (0.519)	0.211 (0.508)
Rural digital infrastructure	0.000* (0.000)	0.000*** (0.000)
Control variables	YES	YES
Constant	−1.804* (1.008)	−2.809*** (0.972)
First stage F value	273.62	
N	8710	8710

Note: ***,** and * indicate significance at the 1%,5% and 10% levels, respectively. The standard errors are clustered at the household level and are reported in parentheses.

### 4.5. Heterogeneity results

To examine the heterogeneity of the impact of migrant working experience on rural households’ digital financial participation across groups, this paper regresses the sample into groups with respect to the types of work performed during migrant work, long-tail groups, and regional differences, respectively.

#### 4.5.1. Types of work.

The type of work during migrant work has an important impact on the ability of migrant workers to effectively accumulate experience, knowledge, and contacts. It is generally recognized that people with formal work or entrepreneurial experience typically not only have higher income levels, but are also more likely to have access to a wealth of information and asset management skills [40], factors that contribute to their better participation in digital financial markets.

Table 8 reports the impact of migrants’ formal work experience and migrants’ entrepreneurial experience on rural households’ digital financial participation. The results in columns (1) and (3) show that the coefficients of the impact of migrant formal work experience on the probability of digital financial participation and the breadth of participation of rural households are both significantly positive, suggesting that migrant formal work experience has a greater facilitating effect. The reason is that engaging in formal work usually has a more stable wage income and a clearer compensation system, which makes migrant farm households more confident in investing and borrowing on digital finance platforms and enhances their motivation to participate in digital finance. The results in columns (2) and (4) show that the effect of migrant entrepreneurship experience on the probability of rural households’ digital finance participation is insignificant and the effect on the breadth of digital finance participation is positively significant. The possible explanation is that entrepreneurship itself is characterized by high risk and uncertainty, and many rural households may be wary of digital financial participation because they fear that the use of digital financial products during migrant entrepreneurship may increase financial risk.

**Table 8:** Heterogeneous Results:Type of work.

Variables	Probability of participation in digital finance		Breadth of participation in digital finance	
	Probit		Poisson	
	(1)	(2)	(3)	(4)
Migrant formal work experience	0.066***		0.042*	
	(0.023)		(0.024)	
Migrant entrepreneurship experience		0.216		0.147**
		(0.142)		(0.064)
Control	YES	YES	YES	YES
R^2^/Pseudo R^2^	0.233	0.233	0.139	0.139
N	9096	9096	9096	9096

Note: The coefficient is the marginal effect. ***,** and * indicate significance at the 1%,5% and 10% levels, respectively. The standard errors are clustered at the household level and are reported in parentheses.

#### 4.5.2. Educational level.

The impact of migrant work experience on rural households’ digital financial participation is also likely to vary across farm households’ education levels. This paper examines the sample by dividing it into two groups, those who have not completed compulsory education and those who have completed compulsory education, based on the required number of years of compulsory education in China (9 years).The results in Table 9 show that the marginal contribution of migrant work experience to the probability and breadth of household digital finance participation is greater for farmers who have completed years of compulsory education compared to those who have not completed years of compulsory education. This is because farmers who have completed compulsory education are usually more adaptable and able to acquire digital skills and learn financial literacy more quickly, enabling them to be more willing to engage in digital financial activities and continue to expand the breadth of their participation.

**Table 9:** Heterogeneous results: education level.

Variables	Probability of participation in digital finance		Breadth of participation in digital finance	
Probit		Poisson	
Non-completion of compulsory education	Completion of compulsory education	Non-completion of compulsory education	Completion of compulsory education
(1)	(2)	(3)	(4)
Migrant work experience	0.085***	0.118***	0.087***	0.111***
	(0.012)	(0.032)	(0.013)	(0.037)
Controls variables	YES	YES	YES	YES
N	7913	1183	7913	1183
R^2^/Pseudo R^2^	0.227	0.216	0.138	0.096

Note: The coefficient is the marginal effect. ***,** and * indicate significance at the 1%,5% and 10% levels, respectively. The standard errors are clustered at the household level and are reported in parentheses.

#### 4.5.3 Long-tail group.

In addition, the paper also seeks to understand whether migrant work experiences can facilitate the participation of disadvantaged rural households in digital finance. According to the Long Tail Theory, traditional financial models tend to focus resources on “head” customers at the expense of “tail” customers, and disadvantaged farmers who are excluded from traditional finance are also more likely to be excluded from digital finance. Drawing on existing literature [11], rural households with per capita household incomes below the 2018 Chinese National Poverty Standard Line of 3,200 yuan are defined as the long-tail group. The results in Table 10 show that the coefficients of the impact of migrant work experience on the probability of household digital financial participation and the breadth of participation are larger for the long-tail group than for the non-long-tail group. This suggests that migrant work experience brings more significant marginal improvement effects of cognitive ability enhancement and social network expansion for the long-tail group, which in turn strongly promotes their participation in digital financial activities.

**Table 10:** Heterogeneous Results: Long Tail Group.

Variables	Probability of participation in digital finance		Breadth of participation in digital finance	
	Probit		Poisson	
	Long-tail group	Non-long-tail group	Long-tail group	Non-long-tail group
	(1)	(2)	(3)	(4)
Migrant work experience	0.121*** (0.036)	0.081*** (0.011)	0.104*** (0.037)	0.080*** (0.013)
Controls variables	YES	YES	YES	YES
N	582	8885	582	8885
R^2^/Pseudo R^2^	0.278	0.229	0.192	0.133

Note: The coefficient is the marginal effect. ***,** and * indicate significance at the 1%,5% and 10% levels, respectively. The standard errors are clustered at the household level and are reported in parentheses.

## 5. Mechanism analysis

How does the migrant work experience contribute to rural households’ participation in digital finance? This section will delve further into the channels and mechanisms through which such experiences may have an impact.

### 5.1 Financial literacy

Table 11 reports the results of testing the financial literacy mechanism. Column (1) presents the regression results for the impact of migrant work experience on financial literacy. The coefficient for the financial literacy variable is significantly positive at the 1% significance level, indicating that migrant work experience positively enhances farmers’ financial literacy. The F-value in the first stage is 317.80, far exceeding the empirical value of 10, confirming that the instrumental variables are not weak. Columns (2) and (3) present regression results for the joint effects of migrant work experience and financial literacy on rural households’ probability and breadth of digital financial participation. Both migrant work experience and financial literacy exhibit positive and significant coefficients. The Cragg-Donald Wald F statistic of 87.228 exceeds the Stock-Yogo critical value of 7.03 at the 10% level, confirming no weak instrumentation issues. Furthermore, considering potential violations of the sequential neglectability assumption, this study adopts the research approach from Ima [41,42] to conduct sensitivity tests on the financial literacy mechanism (S3 Fig). The results confirm that the causal inference of the financial literacy mediation effect is valid and robust. These findings collectively demonstrate that migration work experience promotes rural households’ participation in digital finance by enhancing their financial literacy levels.

**Table 11:** Mechanistic test results for financial literacy.

Variables	financial literacy	Probability of participation in digital finance	Breadth of participation in digital finance
	2SLS	IV-Probit	IV-Poisson
	(1)	(2)	(3)
Migrant work experience	0.117*** (0.029)	0.313*** (0.059)	0.280*** (0.053)
financial literacy		0.769*** (0.111)	0.710*** (0.138)
Controls	YES	YES	YES
Constant	−1.077*** (0.335)	−0.362 (0.651)	−1.704** (0.700)
Phase I F-value	317.80		
Cragg-Donald Wald F		87.228	
N	8805	8805	8805
R^2^	0.090		

Note: ***,** and * indicate significance at the 1%,5% and 10% levels, respectively. The standard errors are clustered at the household level and are reported in parentheses.

### 5.2 Social Networks

Table 12 reports the results of testing the social network mechanism. Column (1) presents the regression results for the impact of migrant work experience on social networks. The coefficient for the migrant work experience variable is significantly positive at the 1% statistical level, indicating that migrant work experience significantly expands rural households’ social networks. Columns (2) and (3) present regression results examining the joint effects of migrant work experience and social networks on the probability and breadth of household digital financial participation. Both variables exhibit statistically significant positive coefficients at the 1% level. The Cragg-Donald Wald F-statistic of 267.015 exceeds the Stock-Yogo critical value of 7.03 at the 10% level, confirming the absence of weak instrumental variable issues. Sensitivity analysis (S3 Fig) on the social network mechanism confirms the validity and robustness of the causal inference regarding the mediating effect of social networks. These findings demonstrate that migrant work experience promotes rural household participation in digital finance by expanding their social networks.

**Table 12:** Mechanistic test results of social networks.

Variables	Social network	Probability of participation in digital finance	Breadth of participation in digital finance
	2SLS	IV-Probit	IV-Poisson
	(1)	(2)	(3)
Migrant work experience	0.091***	0.346***	0.273***
	(0.013)	(0.060)	(0.053)
Social network		0.873***	0.757***
		(0.220)	(0.143)
Controls	YES	YES	YES
Constant	−0.450*** (0.138)	−1.123* (0.660)	−2.762*** (0.647)
Phase I F-value	312.73		
Cragg-Donald Wald F		267.015	
N	8760	8749	8749
R^2^	0.611		

Note: ***,** and * indicate significance at the 1%,5% and 10% levels, respectively. The standard errors are clustered at the household level and are reported in parentheses.

## 6. Conclusion and policy recommendations

### 6.1. Conclusion

Insufficient digital financial participation of rural households largely hinders the development of rural inclusive finance and farmers’ access to the dividends of digital economic development. Starting from the new perspective of migrant work experience, this study examines the impact of migrant work experience on rural households’ digital financial participation based on the 2019 China Household Finance Survey (CHFS) data. The study found that migrant work experience is an important influence on rural households’ digital financial participation, significantly contributing to the probability of digital financial participation and increasing the breadth of participation. Heterogeneity analysis finds that migrant work experience promotes digital financial participation of rural households with migrant formal work experience, migrant entrepreneurial experience, completion of compulsory education, and long-tail groups more. Mechanism analysis finds that migrant work experience can influence rural households’ digital financial participation by improving their financial literacy and broadening their social networks.

### 6.2. Policy recommendations

Based on the above research findings, this article puts forward the following policy recommendations.

First, while encouraging farmers to seek employment outside their hometowns, the government should focus on optimizing the employment environment and providing robust support to farming households. For instance, this could involve expanding formal employment channels through vocational skills training, or supporting independent entrepreneurship through business guarantees and subsidies. In addition, For migrant working farmers with a low level of education, training in the basics of digital finance is provided on a case-by-case basis, and personalized digital finance guidance services are provided to enhance their willingness to participate in digital finance. Financial institutions should develop low-threshold, flexible and diverse digital financial products to meet the financial needs of low-income migrant working farmers in different scenarios, based on their actual needs. At the same time, the leading role of farmers with high levels of education should be utilized, and these farmers should be encouraged to serve as volunteers or tutors for digital financial education, so as to teach digital financial skills to other farmers and enhance the willingness of farmers with low levels of education to participate in digital finance.

Secondly, the Government should further strengthen education and training in rural financial knowledge and expand the social network of migrant working farmers. The government can collaborate with financial institutions to establish dedicated online communication platforms for migrant workers or regularly organize offline forums, providing spaces for them to exchange work and life experiences as well as financial knowledge. This approach enables them not only to share insights on using digital finance face-to-face but also to foster mutual trust and connections, expand their social networks, and ultimately boost the entire group’s enthusiasm for adopting digital financial services.

## Supporting information

S1 Table Balance tests of explanatory variables before and after propensity score matching. (DOCX)

S2 Table Descriptive statistics for panel data. (DOCX)

S3 Fig Sensitivity test. (DOCX)

## References

[1] Huang YP, Huang Z. The development of digital finance in China: Present and future. China Economics Quarterly. 2018;17(4):1489–502. doi: 10.13821/j.cnki.ceq.2018.03.09

[2] Li J, Wu Y, Xiao JJ. The impact of digital finance on household consumption: Evidence from China. Economic Modelling. 2020;86:317–26. doi: 10.1016/j.econmod.2019.09.027

[3] Xie XL, Shen Y, Zhang HX, et al. Can digital finance promote entrepreneurship? - Evidence from China. China Economics Quarterly. 2018;17(4):1557–80. doi: 10.13821/j.cnki.ceq.2018.03.12

[4] Liu S, Koster S, Chen X. Digital divide or dividend? The impact of digital finance on the migrants’ entrepreneurship in less developed regions of China. Cities. 2022;131:103896. doi: 10.1016/j.cities.2022.103896

[5] Meng K, Xiao JJ. Digital finance and happiness: evidence from China. Information Technology for Development. 2022;29(1):151–69. doi: 10.1080/02681102.2022.2097622

[6] Sun Y, Tang X. The impact of digital inclusive finance on sustainable economic growth in China. Finance Research Letters. 2022;50:103234. doi: 10.1016/j.frl.2022.103234

[7] Chibba M. Financial Inclusion, Poverty Reduction and the Millennium Development Goals. Eur J Dev Res. 2009;21(2):213–30. doi: 10.1057/ejdr.2008.17

[8] Das S, Chatterjee A. Impacts of ICT and digital finance on poverty and income inequality: a sub-national study from India. Information Technology for Development. 2023;29(2–3):378–405. doi: 10.1080/02681102.2022.2151556

[9] Li C, Zhang L. The use of ICT, financial transaction costs and rural households’ digital financial participation: An investigation based on the three dimensions of distance, density and human relationship cost. Journal of Nanjing Agricultural University (Social Sciences Edition). 2023;23(02):168–77. doi: 10.19714/j.cnki.1671-7465.20221221.001

[10] Pan S, Wei J, Hu S. Internet finance and household credit constraint mitigation: Evidence based on the heterogeneity of risk preference. Economic Review. 2020;:149–62. doi: 10.19361/j.er.2020.03.14

[11] Zhang L, Li C, Wang R. Financial literacy and rural households’ response to digital financial behavior: Micro evidence from rural household survey in four provinces. Chinese Rural Economy. 2021;437(05):83–101. doi: 10.20077/j.cnki.11-1262/f.2021.05.005

[12] Wen T, Liu Y. Digital literacy, financial knowledge with farmers’ response to digital financial behavior. Research on Financial and Economic Issues. 2023;02:50–64. doi: 10.19654/j.cnki.cjwtyj.2023.02.005

[13] Lo Prete A. Digital and financial literacy as determinants of digital payments and personal finance. Economics Letters. 2022;213:110378. doi: 10.1016/j.econlet.2022.110378

[14] Han L, Xiao JJ, Su Z. Financing knowledge, risk attitude and P2P borrowing in China. Int J Consumer Studies. 2018;43(2):166–77. doi: 10.1111/ijcs.12494

[15] Su L, Peng Y, Kong R, Chen Q. Impact of E-Commerce Adoption on Farmers’ Participation in the Digital Financial Market: Evidence from Rural China. JTAER. 2021;16(5):1434–57. doi: 10.3390/jtaer16050081

[16] Zhou N, Zhang L. Traditional financial foundation, digital endowment and rural digital financial inclusion. Finance and Trade Research. 2022;33(12):49–58.

[17] Liu C, Feng T, Li Y. Network infrastructure construction, digital inclusive finance and the digital divide: an analysis of policy effects based on the creation of “broadband China demonstration cities. Financial Science. 2022;417(12):103–16.

[18] Wang X. Epidemic shocks and rural households’ digital financial behavior-micro evidence from the Jiangsu Rural Household Finance Survey. Finance and Trade Research. 2022;33(06):65–79. doi: 10.19337/j.cnki.34-1093/f.2022.06.006

[19] Hertwig R, Barron G, Weber EU, Erev I. Decisions from experience and the effect of rare events in risky choice. Psychol Sci. 2004;15(8):534–9. doi: 10.1111/j.0956-7976.2004.00715.x 15270998

[20] Malmendier U, Tate G, Yan J. Overconfidence and Early‐Life Experiences: The Effect of Managerial Traits on Corporate Financial Policies. The Journal of Finance. 2011;66(5):1687–733. doi: 10.1111/j.1540-6261.2011.01685.x

[21] Schmid S, Baldermann S. CEOs’ International Work Experience and Compensation. Manag Int Rev. 2021;61(3):313–64. doi: 10.1007/s11575-021-00444-z

[22] Long W, Tian GG, Hu J, Yao D (Troy). Bearing an imprint: CEOs’ early-life experience of the Great Chinese Famine and stock price crash risk. International Review of Financial Analysis. 2020;70:101510. doi: 10.1016/j.irfa.2020.101510

[23] Hui BPH, Ngai P, Qiu JL, Koo A. Having Less But Giving More: Work Experience and Prosocial Behavior of Chinese Working-Class Youth. Youth & Society. 2019;52(8):1582–601. doi: 10.1177/0044118x19840239

[24] Chen G, Qi Y, Liu F, Xing F. Taxation officers’ grassroots work experience and tax performance: Evidence from China. Journal of Asian Economics. 2022;80:101463. doi: 10.1016/j.asieco.2022.101463

[25] Zhou G, Tan H, Li L. Is outworking experience beneficial to returning migrant workers’ entrepreneurship?. Economics (Quarterly). 2017;16(02):793–814. doi: 10.13821/j.cnki.ceq.2017.01.15

[26] James Holtum P, Irannezhad E, Marston G, Mahadevan R. Business or Pleasure? A Comparison of Migrant and Non-Migrant Uber Drivers in Australia. Work, Employment and Society. 2021;36(2):290–309. doi: 10.1177/09500170211034741

[27] Lusardi A, Mitchell OS, Curto V. Financial Literacy among the Young. Journal of Consumer Affairs. 2010;44(2):358–80. doi: 10.1111/j.1745-6606.2010.01173.x

[28] Twumasi MA, Jiang Y, Ding Z, Wang P, Abgenyo W. The Mediating Role of Access to Financial Services in the Effect of Financial Literacy on Household Income: The Case of Rural Ghana. Sage Open. 2022;12(1). doi: 10.1177/21582440221079921

[29] Yin Z, Song Q, Wu Y. Financial knowledge, investment experience and household asset choice. Economic Research. 2014;49(04):62–75.

[30] Lu E. Out-of-Province Work Experience and Rural Household Financial Asset Choice. Journal of Zhongnan University of Economics and Law. 2020;:127–35. doi: 10.19639/j.cnki.issn1003-5230.2020.0010

[31] Jiankui X, Longyao Z, Danmei N. Research on the same group effect in the decision-making of farmers’ use of digital inclusive finance. Journal of Nanjing Agricultural University (Social Sciences Edition). 2023;23(06):176–86. doi: 10.19714/j.cnki.1671-7465.2023.0092

[32] Dershem L, Gzirishvili D. Informal social support networks and household vulnerability: Empirical findings from Georgia. World Development. 1998;26(10):1827–38. doi: 10.1016/s0305-750x(98)00085-0

[33] Olliff L, Baak M, Baddeley M, Lino Lejukole J, Munyonge E, Saidi I, et al. “We will start building from that”: Social capital, social networks and African migrants’ job‐seeking experiences in Australia. Aust J Social Issues. 2022;57(3):725–42. doi: 10.1002/ajs4.205

[34] Baron RM, Kenny DA. The moderator-mediator variable distinction in social psychological research: conceptual, strategic, and statistical considerations. J Pers Soc Psychol. 1986;51(6):1173–82. doi: 10.1037//0022-3514.51.6.1173 3806354

[35] van Rooij M, Lusardi A, Alessie R. Financial literacy and stock market participation. Journal of Financial Economics. 2011;101(2):449–72. doi: 10.1016/j.jfineco.2011.03.006

[36] Zhang C, Sun Y, Hu R, Yang F, Shen X. The impact of rural-urban migration experience on fertilizer use: Evidence from rice production in China. Journal of Cleaner Production. 2021;280:124429. doi: 10.1016/j.jclepro.2020.124429

[37] Shi X, Cui L, Huang Z, Zeng P, Qiu T, Fu L, et al. Impact of internal migration on household energy poverty: Empirical evidence from rural China. Applied Energy. 2023;350:121780. doi: 10.1016/j.apenergy.2023.121780

[38] Bian Y. Bringing Strong Ties Back in: Indirect Ties, Network Bridges, and Job Searches in China. American Sociological Review. 1997;62(3):366. doi: 10.2307/2657311

[39] Freedom M. Lineage organization in southeastern China. Oxfordshire: Berg. 2004.

[40] Dustmann C, Kirchkamp O. The optimal migration duration and activity choice after re-migration. Journal of Development Economics. 2002;67(2):351–72. doi: 10.1016/s0304-3878(01)00193-6

[41] Imai K, Keele L, Tingley D. A general approach to causal mediation analysis. Psychol Methods. 2010;15(4):309–34. doi: 10.1037/a0020761 20954780

[42] Imai K, Keele L, Tingley D, Yamamoto T. Unpacking the Black Box of Causality: Learning about Causal Mechanisms from Experimental and Observational Studies. Am Polit Sci Rev. 2011;105(4):765–89. doi: 10.1017/s0003055411000414

