# Supplementary material for: The impact of migrant work experience on rural households’ participation in digital finance: Evidence from China
Source: PLoS One. 2025 Nov 21;20(11):e0337525. doi: 10.1371/journal.pone.0337525 (PMC12637937; doi:10.1371/journal.pone.0337525)
Supplement: S1 Table — (DOCX) [file pone.0337525.s001.docx]

The equilibrium test is to examine whether the differences between the experimental and control groups in the control variables are eliminated after matching. It is generally considered that the matching results compound the equilibrium hypothesis when the B-value is less than 25% and the R-value is 0.5-2 in the equilibrium test. S1 Table reports the equilibrium test results after matching using the four matching methods. the B-value is greater than 25% before matching, which indicates that there is some difference between the experimental group and the control group. We used four matching methods of nearest neighbor matching (K=3), radius matching, kernel matching and local linear regression matching, respectively, and after matching, the Pseudo R2, LR value, mean deviation, and B-value of the four methods all decreased substantially, which can be judged that the matching results are consistent with the equilibrium hypothesis.

**S1 Table** Balance tests of explanatory variables before and after propensity score matching

| Matching Methods | Pseudo R^2^ | LR chi^2^ | P Value | Mean Deviation(%) | Median Deviation(%) | B Value | R Value |
| --- | --- | --- | --- | --- | --- | --- | --- |
| Pre-match | 0.055 | 442.17 | 0.000 | 13.2 | 13.2 | 60.6 | 0.92 |
| Nearest-Neighbor Matching | 0.001 | 4.71 | 0.994 | 1.9 | 1.8 | 8.0 | 0.98 |
| Radius Matching | 0.000 | 1.48 | 1.000 | 0.8 | 0.5 | 4.5 | 0.89 |
| Nuclear matching | 0.001 | 2.97 | 1.000 | 1.4 | 1.2 | 6.3 | 1.18 |
| Local linear matching | 0.002 | 6.88 | 0.961 | 2.2 | 1.9 | 9.6 | 0.99 |
