# Supplementary material for: The impact of migrant work experience on rural households’ participation in digital finance: Evidence from China
Source: PLoS One. 2025 Nov 21;20(11):e0337525. doi: 10.1371/journal.pone.0337525 (PMC12637937; doi:10.1371/journal.pone.0337525)
Supplement: S2 Table — (DOCX) [file pone.0337525.s002.docx]

**S2 Table** Descriptive statistics for panel data

| Variables | Full sample | | | No migrant working experience | | | migrant working experience | | | Mean Difference |
| --- | --- | --- | --- | --- | --- | --- | --- | --- | --- | --- |
|  | Obs | Mean | Std. | Obs | Mean | Std. | Obs | Mean | Std. |  |
| Participate in digital finance | 9796 | 0.215 | 0.411 | 8406 | 0.197 | 0.398 | 1390 | 0.322 | 0.467 | -0.12^***^ |
| Breadth of participation | 9796 | 0.246 | 0.498 | 8406 | 0.225 | 0.480 | 1390 | 0.371 | 0.579 | -0.15^***^ |
| migrant working experience | 9796 | 0.142 | 0.349 | 8406 | 0 | 0 | 1390 | 1 | 0 | -1.00 |
| Gender | 9796 | 0.896 | 0.306 | 8406 | 0.885 | 0.319 | 1390 | 0.958 | 0.202 | -0.07^***^ |
| Age | 9796 | 55.209 | 11.078 | 8406 | 55.742 | 10.924 | 1390 | 51.987 | 11.456 | 3.75^***^ |
| Education | 9796 | 7.278 | 3.309 | 8406 | 7.152 | 3.373 | 1390 | 8.041 | 2.772 | -0.89^***^ |
| Marriage | 9796 | 0.902 | 0.297 | 8406 | 0.900 | 0.300 | 1390 | 0.916 | 0.278 | -0.02^*^ |
| Health | 9796 | 0.754 | 0.431 | 8406 | 0.745 | 0.436 | 1390 | 0.809 | 0.393 | -0.06^***^ |
| Home | 9796 | 0.968 | 0.176 | 8406 | 0.967 | 0.180 | 1390 | 0.977 | 0.150 | -0.01^**^ |
| Size | 9796 | 3.542 | 1.685 | 8406 | 3.508 | 1.683 | 1390 | 3.747 | 1.689 | -0.24^***^ |
| Older | 9796 | 0.192 | 0.319 | 8406 | 0.196 | 0.324 | 1390 | 0.163 | 0.292 | 0.03^***^ |
| Child | 9796 | 0.108 | 0.161 | 8406 | 0.103 | 0.159 | 1390 | 0.135 | 0.173 | -0.03^***^ |
| Income | 9796 | 5.504 | 13.023 | 8406 | 5.291 | 12.934 | 1390 | 6.795 | 13.483 | -1.50^***^ |
| Assets | 9796 | 39.632 | 90.506 | 8406 | 38.782 | 92.896 | 1390 | 44.774 | 74.259 | -5.99^**^ |
| Commerce | 9796 | 0.094 | 0.292 | 8406 | 0.088 | 0.284 | 1390 | 0.129 | 0.336 | -0.04^***^ |
| Pergdp | 9796 | 10.931 | 0.344 | 8406 | 10.934 | 0.346 | 1390 | 10.919 | 0.328 | 0.01 |
| East | 9796 | 0.327 | 0.469 | 8406 | 0.334 | 0.472 | 1390 | 0.287 | 0.453 | 0.05^***^ |
| West | 9796 | 0.369 | 0.483 | 8406 | 0.367 | 0.482 | 1390 | 0.382 | 0.486 | -0.02 |
