# Supplementary material for: The impact of migrant work experience on rural households’ participation in digital finance: Evidence from China
Source: PLoS One. 2025 Nov 21;20(11):e0337525. doi: 10.1371/journal.pone.0337525 (PMC12637937; doi:10.1371/journal.pone.0337525)
Supplement: S3 Fig — (DOCX) [file pone.0337525.s003.docx]

To further examine the causal relationship between financial literacy and the mediating effect of social networks, this study conducted sensitivity tests following Ima's research approach [34][35]. The core methodology involves relaxing the assumption of ρ=0 by specifying ρ, thereby re-estimating the average causal mediating effect (ACME) under conditions of non-zero correlation.

S3 Fig reports sensitivity test results, illustrating how ACME values change across different levels of sensitivity parameter ρ. Specifically: (a) For financial literacy sensitivity tests, when the sequential ignorability assumption holds (sensitivity test coefficient ρ=0), ACME equals 0.006. If the assumption is violated and sensitivity test parameter ρ is 0.142, the average causal mediation effect is zero. (b) presents the sensitivity test results for social networks. When the sequential ignorability assumption holds (sensitivity test coefficient ρ=0), the average causal mediating effect is 0.006. If the assumption is violated and the sensitivity test parameter ρ is 0.121, the average causal mediating effect is 0. These results indicate that even with some unobserved confounding factors present in this study, which partially violate the sequential ignorability assumption, the causal mediating effects of financial literacy and social networks between immigrant work experience and rural households' digital financial participation remain valid and robust.

| 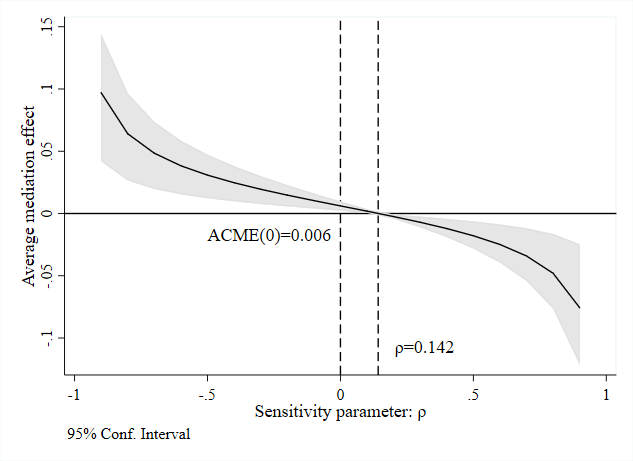 |
| --- |
| **a.** Sensitivity test of financial literacy |
| 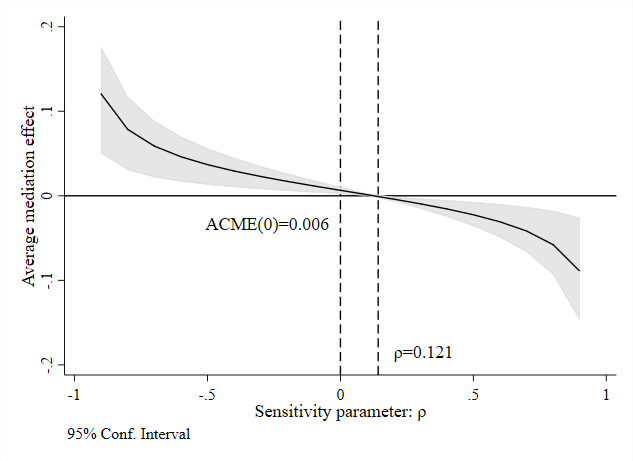 |
| **b.** Social network sensitivity test |
| **S3 Fig** Sensitivity test |
